# Effect of VTMS-Modified TiO_2_ Nanoparticles on CO_2_ Separation Performance of Polysulfone-Based Mixed Matrix Membranes

**DOI:** 10.3390/membranes15120360

**Published:** 2025-11-28

**Authors:** Mustafa Alsaady, Muhammad Faisal Usman, Hafiz Abdul Mannan, Mohamed Amine Ben Ali, Aymn Abdulrahman, Anas Ahmed, Suhaib Umer Ilyas

**Affiliations:** 1 Chemical Engineering Department, University of Jeddah, Jeddah 23890, Saudi Arabiaaabdulrahman@uj.edu.sa (A.A.); 2 Institute of Polymer and Textile Engineering, University of the Punjab, Lahore 54590, Pakistan; faisalusmanbrw@gmail.com; 3 Industrial and Systems Engineering Department, University of Jeddah, Jeddah 23890, Saudi Arabia

**Keywords:** CO_2_ capture, gas separation, mixed matrix membranes (MMMs), nanoparticle dispersion, polysulfone (PSF), surface functionalization, VTMS-modified TiO_2_

## Abstract

Polysulfone (PSF), despite its excellent thermal and mechanical stability, exhibits moderate gas separation performance and poor compatibility with inorganic fillers, resulting in interfacial voids and structural defects. This study addressed these limitations by incorporating vinyltrimethoxysilane (VTMS)-functionalized TiO_2_ nanoparticles into PSF matrix to develop mixed matrix membranes (MMMs) for selective CO_2_/N_2_ separation. Membranes with 1–5 wt.% VTMS@TiO_2_ loadings were fabricated via solution casting, and their gas separation performance was systematically evaluated. VTMS modification enhanced the dispersion and interfacial adhesion of TiO_2_ within the PSF matrix, as confirmed by SEM, FTIR, XRD, and TGA analyses. The 4 wt.% VTMS@TiO_2_ loaded membrane showed optimal performance, with a CO_2_ permeability of 8.48 barrer and a CO_2_/N_2_ selectivity of 26.50 due to stronger polymer–filler interactions and enhanced CO_2_ affinity by VTMS functional groups. This membrane has shown stable transport behavior and favorable CO_2_ selectivity as confirmed by pressure- and temperature-dependent permeation studies. Robeson plot analysis showed that the optimized membrane approached the upper bound, demonstrating a significant improvement over pure PSF. The study confirmed that VTMS-functionalized TiO_2_ enhanced both permeability and selectivity through improved filler dispersion, interfacial integrity, and CO_2_ affinity.

## 1. Introduction

The exponential increase in atmospheric CO_2_ concentration, largely due to fossil fuel combustion and industrial emissions, has led to pressing environmental concerns such as global warming and ocean acidification [1]. According to the International Energy Agency (IEA) report, global CO_2_ emissions reached an all-time high of 36.8 gigatons in 2022, indicating an urgent need for improved CO_2_ capture techniques [2]. Membrane-based gas separation has emerged as a promising, energy-efficient, and environmentally friendly technology for the selective removal of carbon dioxide (CO_2_) from gas mixtures, owing to its simplicity, small footprint, and low operational cost [3]. Membranes turn out to be a reliable approach to CO_2_/N_2_ separation in power plants, gas treatment, and biogas improvement [4]. However, traditional polymeric membranes show a permeability–selectivity trade-off described by Robeson’s Upper Bound, such that materials that have higher permeability exhibit lower selectivity and vice versa. Polymers such as polysulfone (PSF), polyethersulfone (PES), cellulose acetate (CA), and Matrimid exhibit this kind of behavior in gas separation [5,6].

To overcome the limitations demonstrated by polymeric membranes, the innovations in mixed matrix membranes (MMMs) have become a promising means of overcoming these barriers [7]. MMMs refer to a polymer matrix that has inorganic/organic fillers added to it in order to synergize the good features of both phases [8]. Examples of widely used fillers in MMMs are zeolites, carbon molecular sieves, metal–organic frameworks (MOFs), and metal oxide nanoparticles. These fillers offer an excellent intrinsic selectivity and surface area, whereas the polymer matrix supplies high mechanical strength and processability [9,10]. Through a combination of the above components, the composite intends to address the weaknesses of pure polymers. Nevertheless, even though it has been hypothesized that it is superior, MMMs are frequently faced with problems during production, such as insufficient interfacial adhesion, the aggregation of fillers, the appearance of non-selective voids, and thermal instability [11]. Interface imperfections frequently result in performance degradation with a capacity to allow gas to bypass selective regions [12]. Therefore, it is critical to ensure the absence of defects at the filler–polymer matrix interface for the fabrication of efficient MMMs.

Among various polymers used in MMMs, polysulfone (PSF) is a widely employed polymer that is characterized by superior thermal and mechanical properties, and plays a leading role in the production of membranes used in gas separation processes [13]. However, its relatively hydrophobic nature limits its compatibility with many inorganic fillers, particularly hydrophilic metal oxide nanoparticles like titanium dioxide (TiO_2_) [14]. Although the hydrophobicity of PSF is considered a useful property in its operation under humid flue gas streams, the same property lowers its affinity to the hydrophilic nanoparticles. Therefore, surface modification of fillers is required in order to improve dispersion and interfacial adhesion with PSF matrix. TiO_2_ is an attractive filler due to its thermal stability, non-toxicity, chemical inertness, and potential for CO_2_ affinity through surface hydroxyl groups [15]. However, its tendency to aggregate and form weak interfaces within the PSF matrix severely hinders its effectiveness in MMMs [16]. To address this challenge, surface functionalization of fillers is an established strategy to enhance polymer–filler interactions and ensure uniform dispersion. Functional groups such as amines, carboxyls, and silanes can form covalent or hydrogen bonds with the polymer, thereby mitigating phase separation and improving gas transport behavior [17].

Scientific findings prove that modified fillers can increase the effectiveness of gas separation [18]. For example, a study was conducted by using supercritical CO_2_ to silanize nanosilica, which was then mixed with cellulose acetate to make MMMs. Because this silanization method is environmentally friendly, it improved compatibility between the inorganic filler and the polymer, leading to a more uniform membrane with less agglomeration. When compared to the pure CA membranes, the modified CA had reduced hydrogen permeability (by 93%) and a higher CO_2_/H_2_ selectivity [19]. In another study, titanium dioxide nanoparticles were coated and then added to a polymer to increase CO_2_ separation. Modified TiO_2_ nanoparticles could spread better in the polymer, so the MMMs had a much higher CO_2_ permeability and CO_2_/N_2_ selectivity [20]. Mustafa et al. [21] synthesized polyethersulfone and ethylene diamine (EDA) modified TiO_2_ MMMs for CO_2_/CH_4_ separation. The authors concluded that the highest permeability and selectivity were achieved at 5% filler loading. Xin et al. [22] studied amine-functionalization of TiO_2_ and their incorporation in SPEEK matrix. The presence of abundant amine groups on TiO_2_ surface resulted in improved structural, thermal, mechanical, and transport properties. Nguyen studied VTMS modification effect on the properties of polyethylene/TiO_2_ nanocomposites. The authors concluded that this modification has resulted in good dispersibility and adhesion of the modified nanoparticles in the LDPE matrix [23]. These studies demonstrate that surface modification significantly improves filler dispersion and gas separation performance.

In this work, MMMs that combine PSF and VTMS-modified TiO_2_ were successfully prepared and characterized for CO_2_ capture. To the best of the authors’ knowledge, no systematic study is reported on PSF and VTMS-modified TiO_2_-based MMMs for gas separation applications. TiO_2_-based MMMs with other functionalization agents have been reported in the literature, but VTMS-modified TiO_2_-based MMMs are not widely reported. Because VTMS contains methoxy groups that hydrolyze and react with TiO_2_’s surface oxygen, and vinyl groups that bind to PSF’s aromatic rings, it successfully brings together the inorganic and organic phase [24]. Therefore, dual interfacial functionality and CO_2_-philic interfacial chemistry have been introduced in this system by silanol/siloxane bonding and vinyl–aromatic interactions. It is anticipated that with this modification, filler distribution is improved without void formation; CO_2_ sorption selectivity has been enhanced to ultimately improve the separation performance of PSF/VTMS@TiO_2_ MMMs. While MMMs with TiO_2_ have been reported, VTMS-modified TiO_2_ in PSF for CO_2_/N_2_ separation has not been mechanistically evaluated in the prior literature. The modified TiO_2_ particles were prepared by sol–gel surface modification and added to PSF using solution casting to create flat-sheet MMMs with a range of filler loadings. The fabricated membranes were examined with SEM, FTIR, TGA, and XRD to study their morphology, structural strength, thermal behavior, and interfacial properties. Gas permeation analysis was conducted to check the performance of the fabricated mixed matrix membranes for CO_2_ and N_2_ separation applications.

## 2. Materials and Methods

### 2.1. Materials

High-performance thermoplastic polymer Polysulfone (PSF) was obtained from Solvay Advanced Polymers, L.L.C. Analytical grade N-Methyl-2-pyrrolidone (NMP), with a purity of 99.5%, was provided by RCI Labscan Limited, Bangkok, Thailand, and used as the main solvent for the formulation of the membranes. TiO_2_ nanoparticles with a primary particle size of 21 nm were obtained from Sigma Aldrich and used in this study as the inorganic filler. Vinyltrimethoxysilane (VTMS), with at least 99% purity level, was purchased from Shanghai Macklin Biochemical Co., Ltd., Shanghai, China, to increase TiO_2_ compatibility with polymer matrix. High-purity ethanol (99.8%), analytical grade, was obtained from Sigma-Aldrich Co., St. Louis, MO, USA, and employed during the surface modification process. Deionized water used throughout the experiments was supplied by a local laboratory-grade water provider. Finally, carbon dioxide (CO_2_) and nitrogen (N_2_), with a purity of 99.9%, were acquired from a certified local gas supply firm and used for the gas permeation experiments.

### 2.2. Surface Modification of TiO_2_ by VTMS

The TiO_2_ nanoparticles were first dried in a vacuum oven at 100 °C for 24 h to remove adsorbed water. For post-treatment using VTMS, 1 g of the dried nanoparticles was suspended in 40 mL of ethanol through sonication for 10 min. The suspension was stirred at 400 rpm in a sealed vessel and heated to 80 °C for 1 h. Subsequently, 1.03 mL of VTMS was added dropwise over the course of 30 min, and the reaction was left to proceed on at 80 °C for one more day (24 h). When cooled down to room temperature, nanoparticles were separated using centrifugation and carefully washed with ethanol three times in order to remove any residual silane. The prepared VTMS-TiO_2_ nanoparticles were dried under vacuum at 100 °C for 24 h and stored in a desiccator and used at a later stage [18,25]. The schematic illustration of TiO_2_ modification by VTMS is shown in Figure S1 in the Supplementary Data.

### 2.3. Synthesis of PSF/VTMS@TiO_2_ MMMs

A series of mixed matrix membranes was prepared by the solution casting method. In the process of synthesis, the membranes of both pure PSF and PSF-based mixed matrix membrane (MMM) containing different filler loadings of VTMS@TiO_2_ nanoparticle were prepared. N-methyl pyrrolidone (NMP) was used as the solvent for the fabrication of MMMs [26]. The amount of VTMS-modified TiO_2_ nanoparticles ranged from 0 to 0.1 g (0–5%) and was mixed with solvent NMP and subjected to 30 min of ultrasonication to ensure uniform dispersion. Individually, 2 g of PSF was slowly added to the different concentrations of VTMS@TiO_2_-NMP solution, while being kept under continuous magnetic stirring. The mixture was subsequently stirred under ambient temperature overnight to complete dissolution and homogenization. The casting solution was degassed to remove any air bubbles trapped in the solution. In last, the prepared solution was then cast on a clean glass plate using a casting knife. The membranes were dried at 90 °C for 24 h to eliminate residual solvent. After that, thin films were peeled off from the glass plate and kept in zipper bags for further characterization. The compositions of the synthesized membranes, including the amount of VTMS@TiO_2_ and NMP used in each formulation, are summarized in Table 1.

### 2.4. Characterization of VTMS@TiO_2_ and PSF/VTMS@TiO_2_ MMMs

#### 2.4.1. Fourier Transform Infrared Spectroscopy (FTIR)

FTIR analysis was performed using a Shimadzu IR Prestige-21 spectrometer (Kyoto, Japan) in the range of 4000–400 cm^−1^ to identify functional groups and interactions between VTMS-modified TiO_2_ and the PSF matrix. Changes in peak intensity or position confirm successful incorporation and potential bonding between filler and polymer [27]. Additionally, VTMS@TiO_2_ nanoparticles were also analyzed by FTIR analysis to observe the functionalization of TiO_2_ nanoparticles.

#### 2.4.2. Thermogravimetric Analysis (TGA)

Thermal stability of VTMS@TiO_2_ nanoparticles, pure PSF, and PSF/VTMS@TiO_2_ MMMs was assessed using a SDTQ600 analyzer (TA Instruments, New Castle, DE, USA). Nanoparticles, as well as membrane samples of weight (3.5–6.5 mg), were heated from 25 °C to 800 °C at the rate of 10 °C/min ramp under nitrogen gas. The thermogravimetric curve indicated filler influence on decomposition behavior and thermal resistance [28].

#### 2.4.3. Scanning Electron Microscopy (SEM)

Scanning Electron Microscopy (SEM) was used for the surface morphological and dispersion quality analysis of the VTMS-functionalized TiO_2_ in PSF matrix. SEM images were taken at magnification of 7000× using FEI Nova NanoSEM 450 (FEI, Hillsboro, OR, USA) to know the distribution of filler and possible agglomeration. The results of this analysis informed on the interfacial compatibility and structural stability of the produced MMMs [29].

#### 2.4.4. X-Ray Diffraction (XRD)

XRD patterns were recorded using a Cu-Kα radiation (λ = 1.5418 Å) over 2θ = 5 to 80° by using an Equinox 2000 diffractometer (INEL Inc., Stratham, NH, USA) in order to evaluate the crystalline properties of TiO_2_ and its compatibility with PSF matrix. Peak positions and intensity change allowed to infer about structural compatibility [30].

### 2.5. Gas Permeation Analysis

Gas separation performance was determined by CSI 135 gas permeation setup to follow ASTM D-1434. Circular membrane disks (15–20 cm^2^) were placed into a closed cell. The details of permeation setup are described elsewhere [31]. Single gas permeation experiments were conducted in this study, and 3 samples were tested for each membrane. Pure CO_2_ and N_2_ gases (99.9% purity) were passed at different pressures and temperatures, and flow rates were measured with a bubble flow meter. CO_2_ permeability (*P*) was calculated using formula [32]:

(1) $$P=\frac{Q*l}{A*\Delta P}$$

here, *Q* being volumetric flow rate, *l* is membrane thickness, *A* is membrane area, and Δ*P* is pressure difference. Ideal selectivity (α_CO2/N2_) was calculated as the ratio of the permeabilities of CO_2_ and N_2_, respectively [32]:

α_CO_2_/N_2__ = P_CO_2__/P_N_2__ (2)

During the manuscript preparation, a generative artificial intelligence (GenAI) tool, ChatGPT (OpenAI, GPT-4, 2024 version), was used solely to improve the language, grammar, and readability of the text after the authors had completed the scientific writing.

## 3. Results and Discussion

### 3.1. Characterization of VTMS@TiO_2_

**Figure 1:**
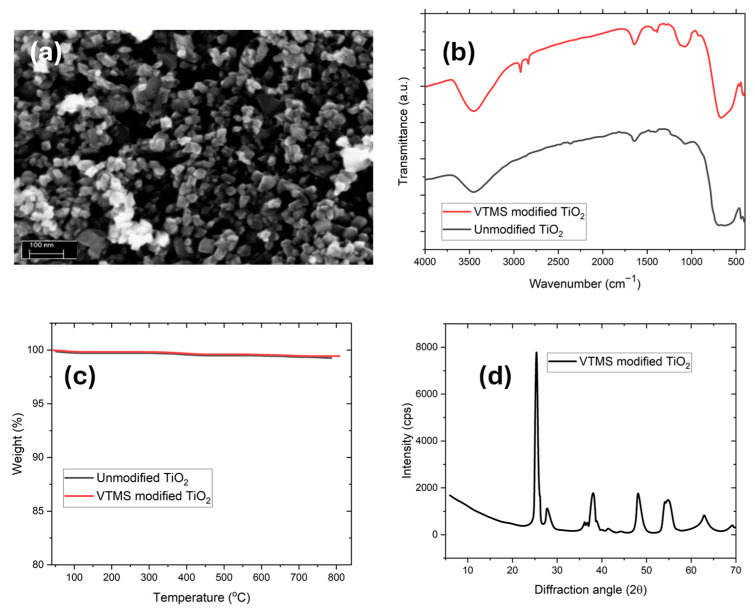
(**a**) SEM image of VTMS-modified TiO_2_ nanoparticles. (**b**) FTIR spectra of unmodified and VTMS-modified TiO_2_ particles. (**c**) TGA curve of unmodified and VTMS-modified TiO_2_ particles. (**d**) XRD pattern of VTMS-modified TiO_2_ nanoparticles.

The morphological characteristics of VTMS-functionalized TiO_2_ nanoparticles were investigated using Scanning Electron Microscopy (SEM), as shown in Figure 1a. The image shows agglomeration-free, nanoscale particles exhibiting quasi-spherical to slightly irregular morphologies. The average particle size is below 100 nm, with uniform particle dispersion across the observed field. The clean and well-defined particle boundaries suggest effective surface functionalization with VTMS, which likely contributed to enhanced steric hindrance and reduced particle–particle interactions. The absence of significant agglomerates demonstrates that the modification process improved the colloidal stability of TiO_2_, which is essential for further applications in polymer nanocomposites and mixed matrix systems [33].

Fourier Transform Infrared (FTIR) spectroscopy was performed to confirm the chemical modification of TiO_2_ nanoparticles with VTMS in Figure 1b. The spectrum of unmodified TiO_2_ primarily shows broad absorption bands below 800 cm^−1^, corresponding to Ti-O-Ti stretching vibrations [34]. Upon VTMS modification, new absorption bands emerge between 1000 and 1200 cm^−1^, which are attributed to Si-O-Si and Si-O-Ti bonds, which is evidence of successful salinization [23]. More peaks in the range of 2850–2950 cm^−1^ refer to aliphatic C-H stretching vibrations of the vinyl groups of VTMS [35]. These spectral characteristics confirm covalent coupling of silane moiety onto the TiO_2_ surface, thus confirming the surface functionalization protocol [36].

Thermogravimetric Analysis (TGA) was used to evaluate the thermal properties of unmodified and VTMS-modified TiO_2_ nanoparticles shown in Figure 1c. The TiO_2_ in its original form showed minimal weight loss between the temperature range of 25 °C to 700 °C, which illustrates its inherent stability towards temperature changes. In comparison, the VTMS-modified TiO_2_ lost weight slightly (1–2%), with most of the weight loss taking place within the range of 200–500 °C. This mass loss is explained by the thermal decomposition of the organic silane functional groups that are grafted on the surface of the TiO_2_. Additionally, TiO_2_ is a thermally stable inorganic oxide, and its functionalization with VTMS does not affect its structural stability but enhances surface chemistry and interfacial compatibility with the polymer matrix. The thermal decomposition profile confirms the presence of organic content while demonstrating that the modified nanoparticles retain high thermal stability, essential for processing in high-temperature membrane fabrication or catalytic applications [37].

X-ray Diffraction (XRD) analysis was conducted to determine the crystalline phase and structural integrity of the VTMS-modified TiO_2_ nanoparticles as depicted in Figure 1d. The diffraction pattern displays distinct peaks at 2θ values around 25.3°, 37.8°, 48.0°, 54.0°, and 62.5°, corresponding to the (101), (004), (200), (105), and (204) planes of anatase TiO_2_, in agreement with JCPDS card No. 21-1272. No additional peaks were detected, indicating phase purity [38]. Furthermore, the crystallinity and peak positions remain unchanged following VTMS treatment, suggesting that the surface modification process does not disrupt the TiO_2_ crystal structure. This preservation of the phase confirms that salinization occurred only on the surface without affecting the bulk structure of the nanoparticles [39]. The particle size of VTMS@TiO_2_ was calculated using the XRD data from the graph to confirm its nanoscale structure, critical for CO_2_ separation applications. The crystallite size (DD) was determined using the Scherrer equation [40,41]. The calculated crystallite size of VTMS@TiO_2_ is 20.35 nm, which confirmed the nanoscale structure of VTMS@TiO_2_. The nanoscale crystallinity enhanced the surface area and is expected to enhance gas interaction, improving the permeability and selectivity of the MMMs for CO_2_ separation.

### 3.2. SEM Analysis of Pure PSF and PSF/VTMS@TiO_2_ MMMs

**Figure 2:**
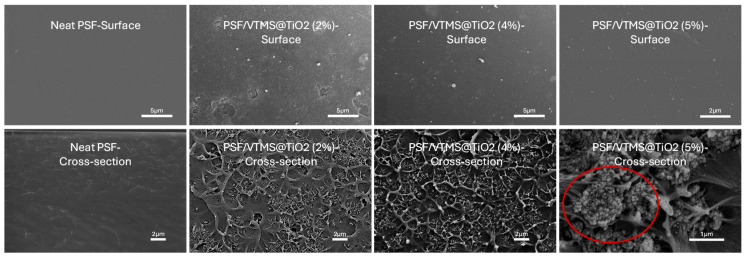
Surface (**top**) and cross-sectional (**bottom**) images of the synthesized MMMs.

The surface and cross-sectional morphological characteristics of pure PSF and PSF-based mixed matrix membranes (MMMs) containing 2 wt.%, 4 wt.%, and 5 wt.% VTMS-functionalized TiO_2_ nanoparticles were analyzed using SEM, as shown in Figure 2. Neat PSF membrane exhibited a smooth and homogeneous surface, which is a dense and defect-free structure. This is a typical structure of unfilled polymeric membranes like PSF. The cross-sectional morphology of neat PSF membrane is homogeneous and non-porous, with no visible phase separation or internal heterogeneities.

The membrane with 2 wt.% VTMS@TiO_2_ in Figure 2 exhibits a relatively smooth polymer surface with irregularly distributed filler particles. While most of the nanoparticles appear embedded within the matrix, several regions display localized micro-aggregates, indicative of partial filler agglomeration. However, no significant phase separation or interfacial voids are visible, suggesting moderate compatibility between the filler and the PSF matrix at this loading. The presence of some clustered domains may affect gas selectivity by introducing less tortuous pathways, although the overall surface remains largely defect-free. The cross-sectional image of 2 wt.% VTMS@TiO_2_ in Figure 2 indicates a dense, continuous morphology, though minor heterogeneity appears at the nanoscale. The nanoparticles are embedded in PSF matrix without forming voids or defects, suggesting strong interfacial adhesion and good polymer–filler compatibility.

In the case of the SEM micrograph of the 4 wt.% VTMS@TiO_2_-filled membrane shown in Figure 2, a markedly improved filler dispersion was observed. The surface is denser and more homogeneously populated with well-embedded nanoparticles, and no visible agglomerates or non-selective voids. Cross-sectional analysis of 4 wt.% VTMS@TiO_2_-filled membrane reveals a slightly more textured internal structure, and the membrane maintains its dense nature, with no evidence of delamination or void formation. This uniform distribution reflects strong interfacial interaction between the VTMS-modified TiO_2_ and the polymer chains, which is a result of enhanced compatibility afforded by the silane coupling agent. The nanofillers appear to be evenly distributed at the microscale, contributing to the formation of a continuous and selective transport pathway for CO_2_ molecules [18]. The cross-sectional SEM of 5 wt.% VTMS@TiO_2_-filled membrane shows significant agglomeration of TiO_2_ particles, highlighted by the red circle. The presence of dense filler clusters and possible non-selective voids around the agglomerates suggests phase separation and poor filler–polymer interaction.

These morphological observations point towards the possible superior gas separation performance for the 4 wt.% membrane, where the balance of high filler loading and excellent dispersion would enhance both permeability and selectivity. The microstructural integrity at this optimal loading level validates the effectiveness of VTMS surface functionalization in minimizing filler aggregation and optimizing polymer–filler interfacial bonding in MMMs designed for CO_2_/N_2_ separation that is also evident from FTIR analysis, as discussed in the following section.

### 3.3. FTIR Analysis of Pure PSF and PSF/VTMS@TiO_2_ MMMs

**Figure 3:**
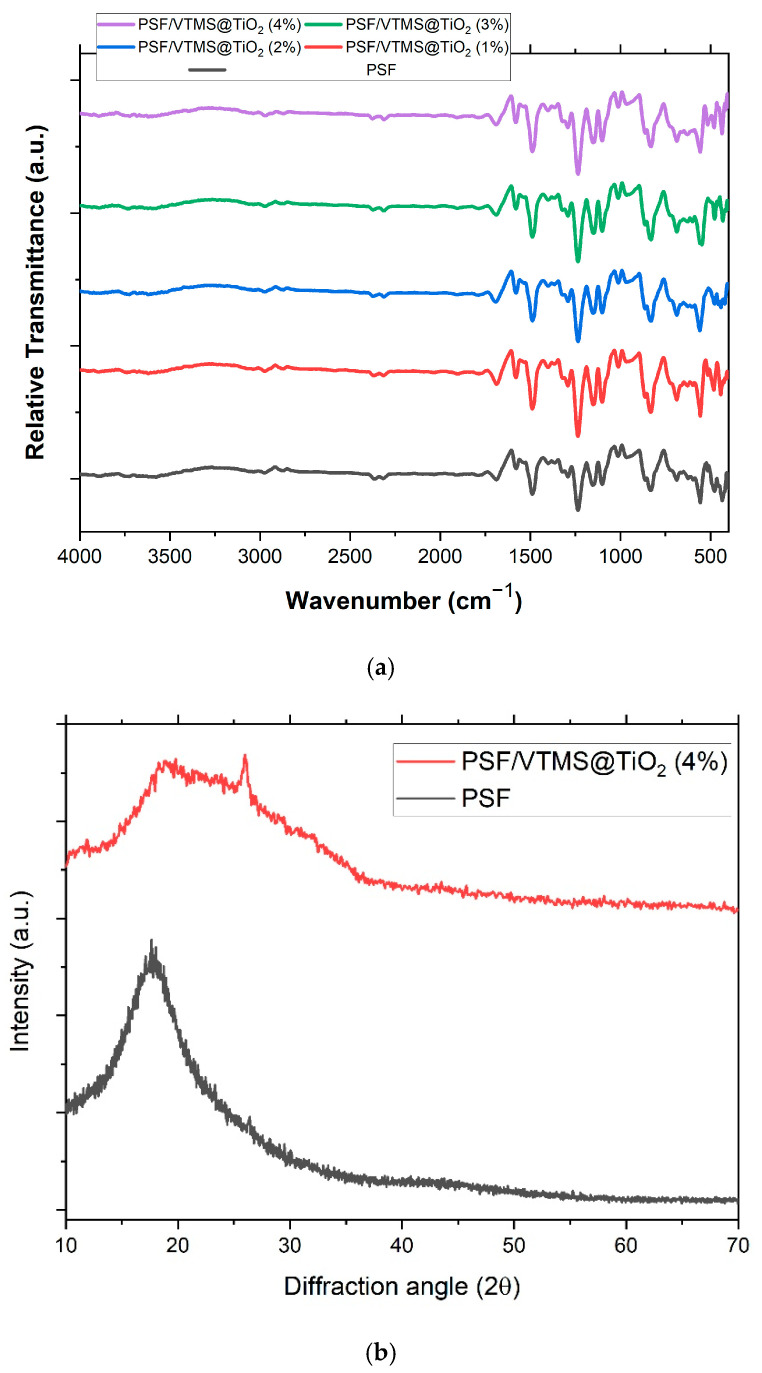
(**a**) FTIR spectra of pure PSF and PSF/VTMS@TiO_2_ MMMs (1–4% Filler). (**b**) XRD patterns of pure PSF and PSF/VTMS@TiO_2_(4%) MMM.

Fourier Transform Infrared (FTIR) spectroscopy was utilized to investigate the chemical interactions between pure polysulfone (PSF) and VTMS-functionalized TiO_2_ nanoparticles across a range of filler concentrations (1–4 wt.%), as shown in Figure 3a. The spectrum of the pure PSF membrane (black curve) exhibited distinct peaks characteristic of its polymer backbone of aromatic C=C stretching at 1586 cm^−1^, sulfone (O=S=O) asymmetric stretching at 1321 cm^−1^, and C-O-C ether linkages at 1231 cm^−1^ and 1156 cm^−1^ [31,42]. Additional signals at 1072 cm^−1^ (S-O/C-H bending) and 823 cm^−1^ (aromatic ring deformation) confirmed the presence of sulfone and aromatic functionalities in the polymer chain [43].

Upon incorporation of VTMS@TiO_2_, the most prominent spectral changes were observed in the membrane containing 4 wt.% filler, which exhibited intensified absorption bands at 1062 cm^−1^ and 970 cm^−1^, corresponding to Si-O-Ti/Si-O-Si linkages and Si-OH groups, respectively [21]. These enhancements suggest a higher degree of chemical bonding between the silane-modified filler and the polymer matrix, indicating successful VTMS grafting and improved interfacial compatibility. A pronounced peak at 690 cm^−1^, attributed to Ti-O lattice vibrations, became more defined with increasing filler concentration, particularly in the 4% sample, confirming the increased presence of TiO_2_ and its good dispersion [44]. Furthermore, the slight increase in intensity of the C-H stretching vibration at 2923 cm^−1^ in the 3% and 4% composites indicates the growing contribution of the VTMS organic moiety. These findings collectively suggest that the 4% VTMS@TiO_2_ membrane demonstrates the most effective integration and surface interaction among all tested compositions. Low loadings of the filler (1–3 wt.%) also showed the same behavioral trend of the spectra but to a reduced extent. The appearance of silanol (Si-OH) and Si-O-Ti features at 1 wt.% was weak, suggesting limited surface coverage or interaction. When loading increased to 2% and 3%, Si-related peaks started to appear in a prominent way which indicates an improvement in the filler distribution and bonding. Notably, among all measured samples, the typical PSF backbone peaks were not affected in position and intensity, thus confirming that the polymer structure does not chemically change and is not destroyed even after the addition of the filled material [45].

The water contact angle measurements shown in Table S1 demonstrate enhanced surface hydrophilicity of the membranes with increasing VTMS@TiO_2_ loading up to 4 wt.%. This trend can be attributed to the introduction of polar silanol and hydroxyl groups as seen in FTIR spectra. However, a slight increase in water contact angle at 5 wt.% indicates filler agglomeration which is also evident in SEM images. The agglomeration leads to reduced exposure of hydrophilic sites and increases surface heterogeneity.

The observed spectral modifications provide evidence to the formation of chemical bonds like Si-O-Ti and Si-O-Si on filler surface, supporting the efficiency of VTMS modification. The stronger silanol (Si-OH) retention also indicates partial condensation that will help interfacial hydrogen bonding or secondary interactions with PSF. These interactions are very important in enhancing performance of the resulting membranes in mechanical, thermal, and separation of gases. Overall, the FTIR results confirm favorable chemically induced TiO_2_ surface modification and successful incorporation into the PSF matrix, with an optimized filler loading of 4 wt.%.

### 3.4. XRD Analysis of Pure PSF and PSF/VTMS@TiO_2_ MMMs

The XRD technique was applied to characterize the phase composition and structure of the pure PSF membrane and MMM having 4 wt.% VTMS@TiO_2_ as shown in Figure 3b. The curve for the pure PSF membrane (black curve) shows a broad and featureless peak at about 2θ ≈ 18.6° and, therefore, it can be concluded that the membrane is mostly amorphous, typical of thermoplastic polysulfone membranes [46]. In contrast, the PSF/VTMS@TiO_2_ (4%) membrane (red curve) shows a significant change in the XRD profile. In addition to the polymer’s amorphous hump, several sharp diffraction peaks appear at 2θ ≈ 25.3°, 37.9°, 48.0°, 54.0°, and 62.6°, which correspond to the (101), (004), (200), (105), and (204) planes of the anatase phase of TiO_2_, respectively, as per JCPDS card No. 21-1272. These peaks confirm that the crystalline structure of TiO_2_ remained intact after VTMS modification and subsequent incorporation into the polymer matrix. The peak at 25.3° (101) is particularly intense and dominant, which is a hallmark of TiO_2_ and confirms its structural stability.

The emergence of these distinct peaks in the MMM spectrum strongly supports the successful dispersion of VTMS@TiO_2_ nanoparticles within the PSF matrix. Moreover, the amorphous part of the PSF’s diffraction pattern did not change, which indicated the polymer’s internal structural integrity and the lack of crystallization of the filler in the polymer matrix. The co-existence of amorphous matrix of PSF and well-defined crystalline structure of TiO_2_ in the composite presents a material that is flexible and rigid at the same time. The increase in the diffraction intensity of the composite membrane points to higher crystallinity from the inorganic filler, which helps to enhance the material’s mechanical strength, thermal stability, and gas permeation properties. From the observed XRD patterns, successful integration of VTMS@TiO_2_ nanoparticles in the PSF matrix can be seen, highlighting the promise of the material for gas separation membranes.

### 3.5. TGA of Pure PSF and PSF/VTMS@TiO_2_ MMMs

**Figure 4:**
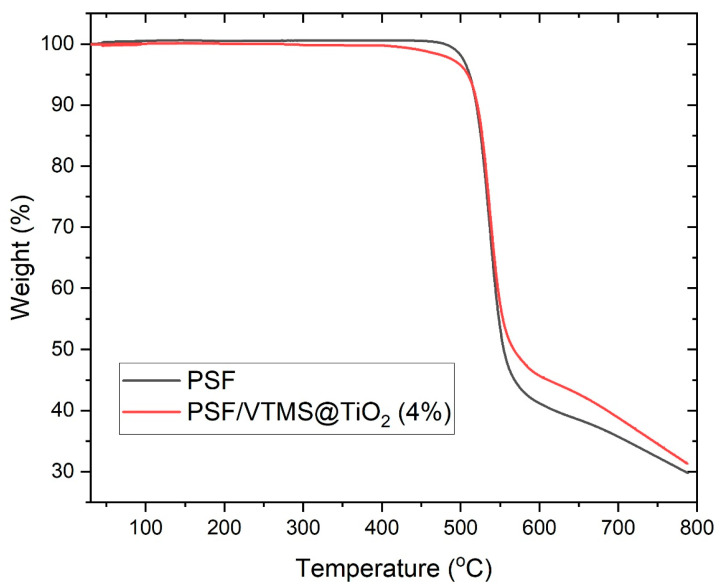
Thermal analysis of pure PSF and PSF/VTMS@TiO_2_ (4%) MMM.

Thermogravimetric Analysis (TGA) was conducted to evaluate the thermal stability and decomposition behavior of the pure polysulfone (PSF) membrane and the PSF-based mixed matrix membrane (MMM) containing 4 wt.% VTMS-functionalized TiO_2_ (VTMS@TiO_2_). The TGA curves, presented in Figure 4, illustrate weight loss (%) as a function of temperature in the range of 30 °C to 800 °C under a nitrogen atmosphere.

The TGA curve of pure PSF (black curve) shows excellent thermal stability up to approximately 500 °C, beyond which a rapid weight loss occurs. This major degradation event corresponds to the thermal decomposition of the PSF backbone, involving scission of sulfone, ether, and aromatic linkages. The onset of degradation (T-onset) is observed around 520 °C, and the maximum decomposition rate occurs near 550–580 °C, leading to a substantial mass loss. By 800 °C, the residual weight is approximately 31%, primarily consisting of carbonaceous char and thermally stable aromatic fragments. On the other hand, the PSF/VTMS@TiO_2_ (4%) membrane (red curve) displays a similar thermal profile but with subtle shifts indicating enhanced thermal resistance. The onset of thermal degradation is slightly delayed, beginning around 530 °C, and the final residue at 800 °C is higher (~35%), which is attributed to the presence of thermally stable TiO_2_ nanoparticles and residual siloxane components from VTMS. The incorporation of VTMS@TiO_2_ contributes to improved char formation and enhanced thermal shielding due to its high decomposition resistance and inorganic nature.

The little increase in onset decomposition temperature and the increased residue content in the MMMs suggest that the filler provides a thermal barrier against heat-catalyzed degradation of the polymer matrix. Additionally, VTMS-functionalized surface could boost interfacial adhesion and restrict the mobility of the chains at high temperatures, adding to thermal stability [18]. The similarity in degradation behavior of pure PSF and PSF/VTMS@TiO_2_ shows that the thermal stability of the composite membrane is still dominated by the PSF matrix, while with introduction of the thermally stable TiO_2_ nanoparticles slightly increases the time scale of the decomposition. Therefore, the slight variation in the TGA curves is mainly due to the low number and high thermal resistance of the VTMS-modified TiO_2_, which proves that the treatment on the surface of the nanoparticles primarily improves the interfacial interaction, with no detrimental effect on the thermal integrity of the nanoparticles or the MMMs. TGA confirmed the incorporation of 4 wt.% VTMS@TiO_2_ in PSF matrix and enhanced the strength of the membrane relating to the thermal robustness, which is beneficial, particularly in high temperature applications like the gas separation processes in toxic industrial environments.

### 3.6. Effect of VTMS@TiO_2_ Loading on Gas Permeation Properties

**Figure 5:**
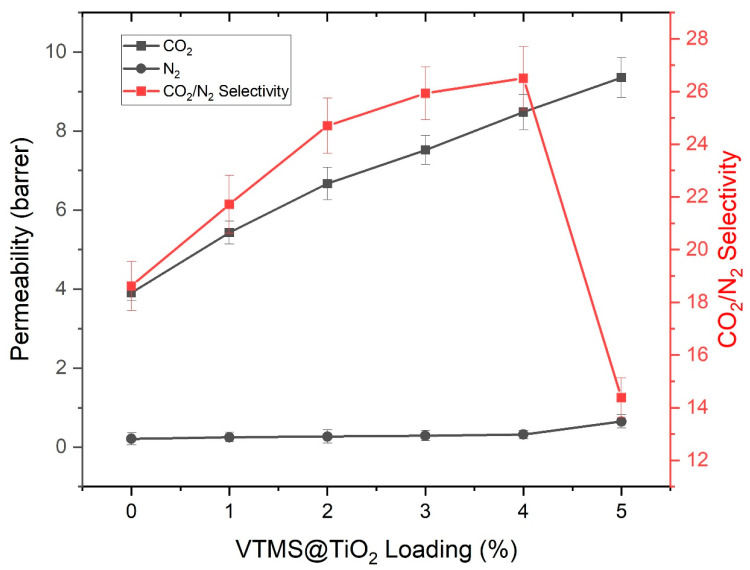
Effect of filler loading on CO_2_ and N_2_ permeability and CO_2_/N_2_ selectivity at 2 bar and 25 °C.

In order to understand the influence of VTMS@TiO_2_ filler loading on the performance of the membranes in terms of CO_2_, N_2_ permeability, and CO_2_/N_2_ selectivity, a graphical representation displayed in Figure 5 was used. The pure PSF membrane (without filler) has shown a CO_2_ permeability of 3.91 barrer and N_2_ permeability of 0.21 barrer, which gives the selectivity as 18.62. Upon incorporating 1 wt.% VTMS@TiO_2_, the CO_2_ permeability increased to 5.43 barrer and the selectivity improved to 21.72, indicating enhanced CO_2_ transport properties due to better gas sorption and diffusion facilitated by the dispersed nanofiller. With 2 wt.% filler loading, CO_2_ permeability further rose to 6.67 barrer, while selectivity increased to 24.70, suggesting improved interfacial compatibility between the filler and polymer matrix. At 3 wt.% loading, the membrane demonstrated a CO_2_ permeability of 7.52 barrer with a selectivity of 25.93. The consistent enhancement in both parameters up to this level is attributed to the uniform dispersion of the VTMS-functionalized TiO_2_ particles as seen in SEM analysis, which likely provided more accessible sorption sites and better chain packing for selective gas transport [43].

The most significant performance was observed at 4 wt.% VTMS@TiO_2_, where the membrane achieved a CO_2_ permeability of 8.48 barrer and the highest CO_2_/N_2_ selectivity of 26.50. This optimum behavior is attributed to the synergistic effects of filler–polymer interactions, enhanced gas affinity of the modified TiO_2_, and the absence of structural defects or agglomeration. The VTMS@TiO_2_ filler improves CO_2_/N_2_ selectivity by introducing CO_2_-philic functional groups in TiO_2_ surface, as observed in FTIR analysis, which enhance CO_2_ sorption. Hence, the selectivity enhancement could be predominantly attributed to sorption in these membranes. In general, gas transport through dense membranes is governed by solution diffusion mechanism. Since TiO_2_ is a nonporous filler, molecular sieving may not be realized in these MMMs. SEM analysis also revealed improved filler dispersion and polymer–filler compatibility. This reduces non-selective voids and creates a more size-selective, solubility-enhanced membrane environment that favors CO_2_ over N_2_. However, at 5 wt.% filler loading, although CO_2_ permeability continued to increase to 9.35 barrer, the selectivity sharply declined to 14.38 due to a significant rise in N_2_ permeability (0.65 barrer). This drop in selectivity is likely due to the onset of filler agglomeration and the formation of non-selective voids at higher loading levels, which disrupts the membrane’s morphological integrity and reduces the selective advantage provided by the filler.

It is worth mentioning that CO_2_ permeability obtained in this research is lower than other PSF-based MMMs filled with highly porous nanostructures (ZIF-8, UiO-66, carbon-based nanostructures, etc.). It is expected that since TiO_2_ is a non-porous filler, it does not add any further microporosity for diffusional transport through the membrane. Therefore, transport of gases in dense PSF matrix is only through the solution-diffusion mechanism, and permeability is highly restricted by the inherent polymer rigidity and low free volume instead of filler porosity. The VTMS modification enhances interfacial compatibility and CO_2_ sorption but cannot create high-throughput channels like porous MOFs or zeolitic fillers. Thus, the permeability is low, but the improvement is based on increased polymer–filler interaction and CO_2_-philic chemistry but not related to increases in microporosity. Thus, 4 wt.% VTMS@TiO_2_ was identified as the optimal loading, balancing both permeability and selectivity, and highlighting the importance of controlling filler dispersion to avoid performance deterioration at higher concentrations.

### 3.7. Effect of Feed Pressure on Gas Permeation Properties

**Figure 6:**
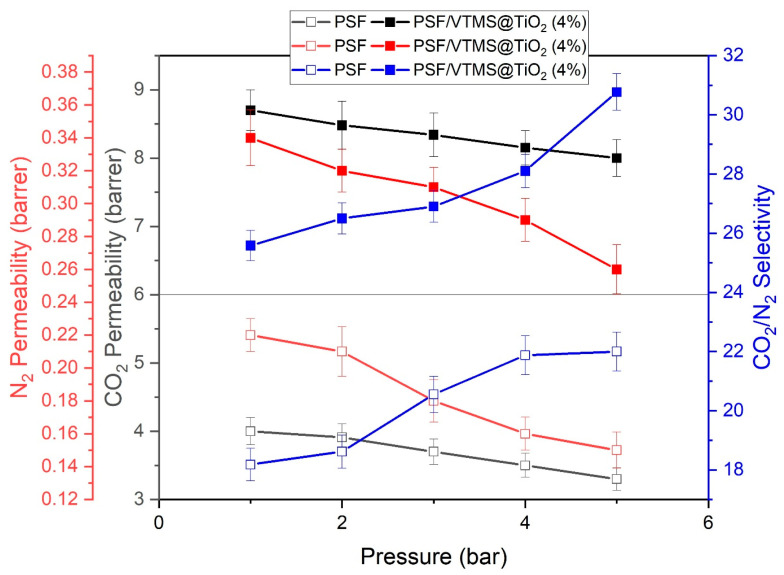
Effect of feed pressure on CO_2_ and N_2_ permeability and CO_2_/N_2_ selectivity at 25 °C.

The impact of feed pressure on CO_2_ and N_2_ permeability as well as CO_2_/N_2_ selectivity was evaluated for the PSF/VTMS@TiO_2_ (4%) mixed matrix membrane (MMM) in Figure 6. As shown in the corresponding plots, the CO_2_ permeability of the membrane shows a slight decline with increasing pressure, decreasing from 8.7 barrer at 1 bar to 8.0 barrer at 5 bar. Similarly, N_2_ permeability also decreases with pressure, from 0.34 barrer to 0.26 barrer across the same range. This pressure-dependent decline in gas permeability is commonly attributed to polymer chain packing and reduced segmental mobility under higher pressure, leading to lower diffusivity [45]. Interestingly, while permeability for both gases decreases, the CO_2_/N_2_ selectivity significantly increases with pressure, rising from 25.58 at 1 bar to 30.77 at 5 bar. This trend indicates that MMM becomes increasingly selective toward CO_2_ at higher pressures, which is a desirable trait in gas separation applications. The enhanced selectivity can be explained by the differential compressibility and kinetic diameters of CO_2_ and N_2_; CO_2_’s higher solubility and smaller kinetic diameter allow it to permeate more favorably, especially under densifying membrane conditions.

Overall, the PSF/VTMS@TiO_2_ (4%) membrane demonstrates excellent performance, maintaining high CO_2_ permeability while achieving superior selectivity at elevated pressures. This behavior makes it a promising candidate for practical CO_2_/N_2_ separation processes, especially where operations occur under moderate to high pressures.

### 3.8. Effect of Feed Temperature on Gas Permeation Properties

**Figure 7:**
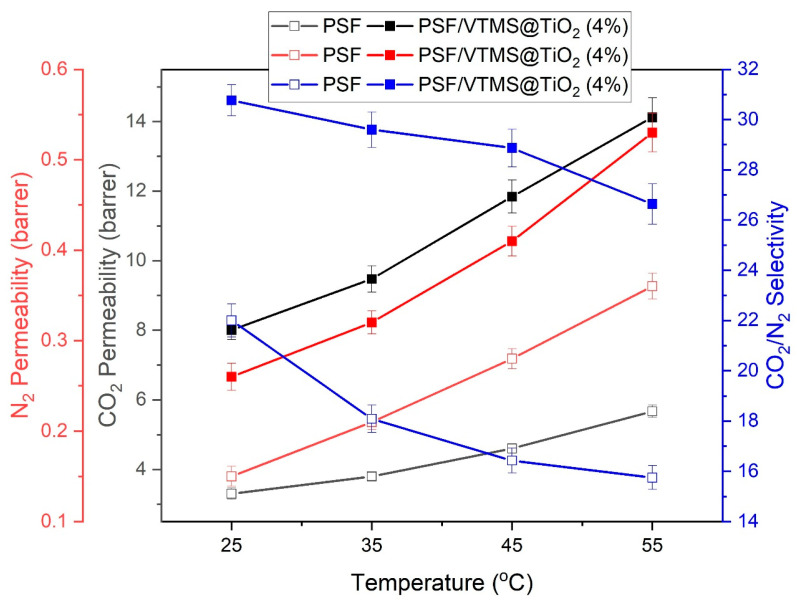
Effect of feed temperature on CO_2_ and N_2_ permeability and CO_2_/N_2_ selectivity at 5 bar.

The effect of feed temperature on the gas transport properties of PSF and PSF/VTMS@TiO_2_ (4%) membranes is illustrated by analyzing the permeability of CO_2_ and N_2_, along with their selectivity ratio (CO_2_/N_2_), across the temperature range of 25–55 °C shown in Figure 7. The incorporation of 4% VTMS-functionalized TiO_2_ nanoparticles significantly enhances both CO_2_ and N_2_ permeability compared to the pure PSF membrane at all temperatures. As temperature increases, the permeability of both gases rises, which is consistent with the increased chain mobility of the polymer matrix and greater diffusivity at higher temperatures [47].

For the PSF/VTMS@TiO_2_ (4%) membrane, CO_2_ permeability increases markedly from 8 barrer at 25 °C to 14.12 barrer at 55 °C, while N_2_ permeability increases from 0.26 to 0.53 barrer over the same range. This rise in permeability is expected due to the thermally activated nature of gas diffusion. However, a key observation is the declining trend in CO_2_/N_2_ selectivity with increasing temperature from 30.77 at 25 °C down to 26.64 at 55 °C, suggesting that the temperature has a more pronounced effect on the diffusion of the less permeable gas (N_2_), thereby reducing the membrane’s ability to discriminate between CO_2_ and N_2_.

Despite the decline in selectivity at higher temperatures, the PSF/VTMS@TiO_2_ (4%) membrane consistently outperforms the unmodified PSF in both permeability and selectivity across the full temperature range. The highest selectivity (30.77) and a reasonable CO_2_ permeability (8 barrer) are observed at 25 °C, indicating this condition as optimal for applications prioritizing separation performance. Therefore, the PSF/VTMS@TiO_2_ (4%) membrane at 25 °C can be considered the optimal sample in this study for efficient CO_2_/N_2_ separation, balancing both high selectivity and adequate permeability. These findings are in line with solution-diffusion mechanism for dense polymeric membranes such as PSF. With the increase in temperature, the influence of sorption decreases due to the exothermic nature of gas–polymer interactions, and the diffusion becomes dominant due to increased segmental mobility [48]. Hence increased permeability and lower selectivity are observed in glassy polymers. Even though the permeability is still less than the highly porous filler systems, yet the VTMS@TiO_2_ filler provides a stable and defect-free interface throughout the temperature range.

### 3.9. Robeson Upper Bound Plot

**Figure 8:**
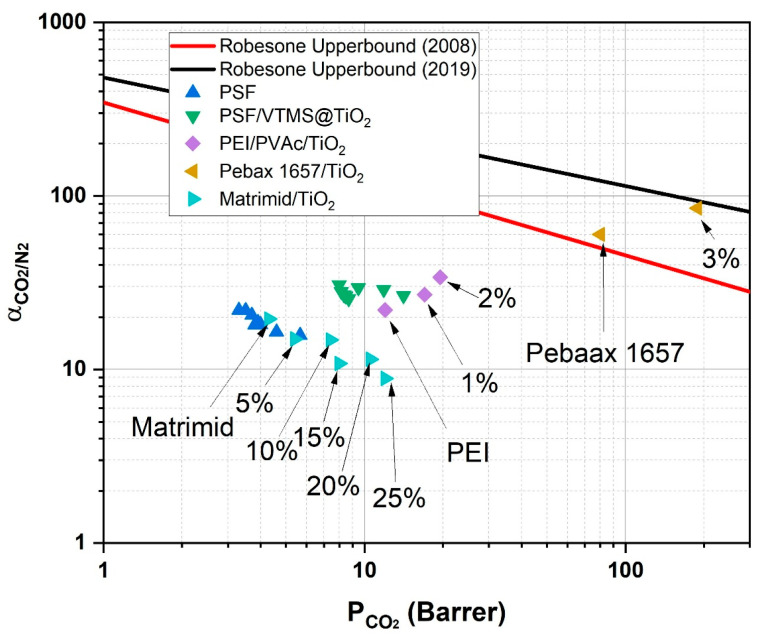
Robeson Upper Bound of PSF and PSF/VTMS@TiO_2_(4%) MMMs.

Figure 8 presents the position of PSF and PSF/VTMS@TiO_2_ (4%) mixed matrix membranes (MMMs) on the Robeson Upper Bound plot for CO_2_/N_2_ separation, offering a comparative assessment of their performance in terms of permeability and selectivity. The Robeson Upper Bound serves as a benchmark for evaluating membrane efficiency, illustrating the typical trade-off between gas permeability and selectivity in polymeric membranes. Ideally, high-performance membranes are those that can simultaneously achieve both high permeability and high selectivity, positioning them closer to or beyond the upper bound line. In this study, the PSF membrane (without any filler) displayed limited separation performance, with a CO_2_ permeability of 3.91 barrer and CO_2_/N_2_ selectivity of 18.62, placing it well below both the 2008 and 2019 Robeson limits. However, incorporation of VTMS-functionalized TiO_2_ nanoparticles into the PSF matrix notably improved membrane performance. Among all tested formulations, the membrane containing 4 wt.% VTMS@TiO_2_ demonstrated the best balance, exhibiting a CO_2_ permeability of up to 14.12 barrer and selectivity reaching 30.77 under optimal operational conditions, including low feed temperature and elevated pressure [49]. Data of three other TiO_2_-filled polymer systems, i.e., Pebax [18], Matrimid [50], and PEI [51] have also been included in the comparison as shown in Figure 8. Matrimid was filled with a higher loading of TiO_2_ which resulted in poor selectivity of this system. PEI/PVAc/TiO_2_ system has shown a superior performance that can be attributed to the presence of both glassy and rubbery polymers in the system along with TiO_2_ nanoparticles. Pebax/TiO_2_ system has a much superior performance as compared to the present study. This is due to the intrinsic superior performance of Pebax polymer. Additionally, the membranes were tested at much higher pressure, i.e., 20 bar. Therefore, higher separation performance was observed.

The enhancement in separation performance with increasing VTMS@TiO_2_ content up to 4 wt.% is attributed to the synergistic effect between the polymer matrix and the well-dispersed, surface-functionalized inorganic filler. The VTMS modification of TiO_2_ improves compatibility with the polymer, enabling uniform dispersion and minimizing interfacial voids, which contribute to a more tortuous and selective pathway for gas molecules. The increased CO_2_ affinity of the filler, coupled with the reduced kinetic diameter of CO_2_ compared to N_2_, further promotes preferential CO_2_ transport. Additionally, other operating conditions like increased feed pressure were seen to increase the selectivity of the membrane without having as much adverse effect on the permeability, indicating the stability and efficiency of the membrane under practical application conditions. Notably, at 4 wt.% loading of VTMS@TiO_2_, the optimal performance was achieved, and its further enhancement to 5 wt.% resulted in a decrease in the selectivity along with an incremental increase in the CO_2_ permeability. The decrease is likely caused by the agglomeration of nanoparticles at high levels, leading to the creation of the non-selective voids, which affect the discriminative ability of the membrane [52]. Thus, PSF/VTMS@TiO_2_ (4%) membrane exhibits a considerable improvement relative to neat PSF, and its location on the Robeson plot is close to the 2019 upper bound.

When compared with recent advances in PSF-based MMMs, the present study is consistent with the literature trend that dense polymer matrices incorporating non-porous fillers typically exhibit moderate permeability but improved selectivity. Higher CO_2_ permeability in PSF membranes is typically achieved by the incorporation of porous fillers such as MOFs or carbon-based materials. However, these MMMs often suffer from poor interfacial compatibility, resulting in decreased selectivity. In contrast, the VTMS-functionalized TiO_2_ used here provides a stable and well-bonded interface, improving selectivity even though the permeability remains moderate. It highlights the promise of VTMS-functionalized TiO_2_ as a promising filler in improving membrane-based CO_2_/N_2_ separation, particularly when used with optimal loading and working environment.

## 4. Conclusions

In this research work, we have successfully synthesized and investigated novel PSF-based mixed matrix membranes (MMMs) containing VTMS-functionalized TiO_2_ nanoparticles for the improved CO_2_/N_2_ separation performance. From FTIR analysis, it was concluded that TiO_2_ nanoparticles have been successfully functionalized and incorporated into the PSF matrix through the appearance of characteristic Si-O-Ti and Si-O-Si bonds, indicating strong interfacial interaction. SEM micrographs of the optimized 4 wt.% VTMS@TiO_2_ membrane revealed homogenous dispersion of nanoparticles without visible agglomeration or phase separation. XRD analysis demonstrated the preservation of the crystalline phase of TiO_2_ within the amorphous PSF matrix, confirming the structural integrity of the nanofiller. TGA further showed improved thermal stability in the MMMs, with the 4 wt.% membrane exhibiting a higher decomposition onset temperature and greater residual weight. Gas permeation results indicated that the 4 wt.% VTMS@TiO_2_ membrane achieved the highest CO_2_ permeability (8.48 barrer) and selectivity (26.50) under standard conditions, with further improvement in selectivity (30.77) observed at 5 bar feed pressure. This study highlights the importance of surface engineering and filler dispersion in overcoming traditional polymer membrane limitations and paves the way for further exploration of silane-based nanocomposites in sustainable gas separation technologies. The VTMS-functionalization approach reported in this work can be extended to porous fillers like MOFs or inorganic–organic hybrid nanostructures to further increase the permeability without losing interfacial compatibility. The enhanced polymer–filler chemistry that has been realized in the present work offers a reliable basis for designing next-generation high-performance MMMs with tailored CO_2_ affinity and reduced interfacial defects. However, extensive studies on mixed gas experiments, solubility and diffusivity, long-term stability, plasticization resistance, and mechanical properties are essential for utilizing the full potential of these membranes.

## Figures and Tables

**Table 1:** Compositions of MMMs and sample codes.

Sr. No.	PSF (g)	VTMS-TiO_2_ (g)	% VTMS-TiO_2_	NMP (g)
1	2	0.0	0	8
2	2	0.02	1	7.98
3	2	0.04	2	7.96
4	2	0.06	3	7.94
5	2	0.08	4	7.92
6	2	0.1	5	7.9

## Data Availability

All data generated during this study have been provided in the manuscript.

## Supplementary Materials

The following supporting information can be downloaded at: https://www.mdpi.com/article/10.3390/membranes15120360/s1. Table S1: Contact angle and thickness of PSF/VTMS@TiO_2_; Table S2: Performance comparison of present work with the literature [18,50,51,53,54]; Table S3: Structures of chemicals used in this study; Figure S1: Schematic illustration of TiO_2_ modification by VTMS.
