# Supplementary material for: Effect of VTMS-Modified TiO2 Nanoparticles on CO2 Separation Performance of Polysulfone-Based Mixed Matrix Membranes
Source: Membranes (Basel). 2025 Nov 28;15(12):360. doi: 10.3390/membranes15120360 (PMC12734554; doi:10.3390/membranes15120360)
Supplement: Supplementary file 1 [file membranes-15-00360-s001.zip › membranes-3861835-supplementary.pdf]

Supplementary Data

Table S1: Contact angle and thickness of PSF/VTMS@TiO_2 MMMs

VTMS@TiO_2 Loading (%)	Contact Angle (°)	Thickness (µm)
0	80.35±2.5	46.32
1	76.59±2.3	59.78
2	74.11±2.2	49.63
3	71.87±2.0	52.18
4	68.48±1.8	56.74
5	70.79±1.8	60.09

Table S2: Performance comparison of present work with literature

Membrane	Test conditions	CO_2 Permeability	Selectivity	Reference
PEI-PVAc/TiO_2	2 bar and 25°C	19.5 GPU	34	[1]
Pebax/dopamine/ polyethyleneimine-grafted TiO_2	0.3 MPa and 20°C	125 barrer	64	[2]
Pebax-1657/3-aminopropyl-diethoxymethylsilane modified TiO_2	20 bar and 25°C	186.6 barrer	84.9	[3]
Matrimid5218/ TiO_2	2–3 bar and 35◦C	5.4 barrer	15	[4]
PSF/ZIF-8	1 bar and 25°C	20.3	12.69	[5]
PSF/VTMS modified TiO_2	5 bar and 25°C	8	30.77	This study

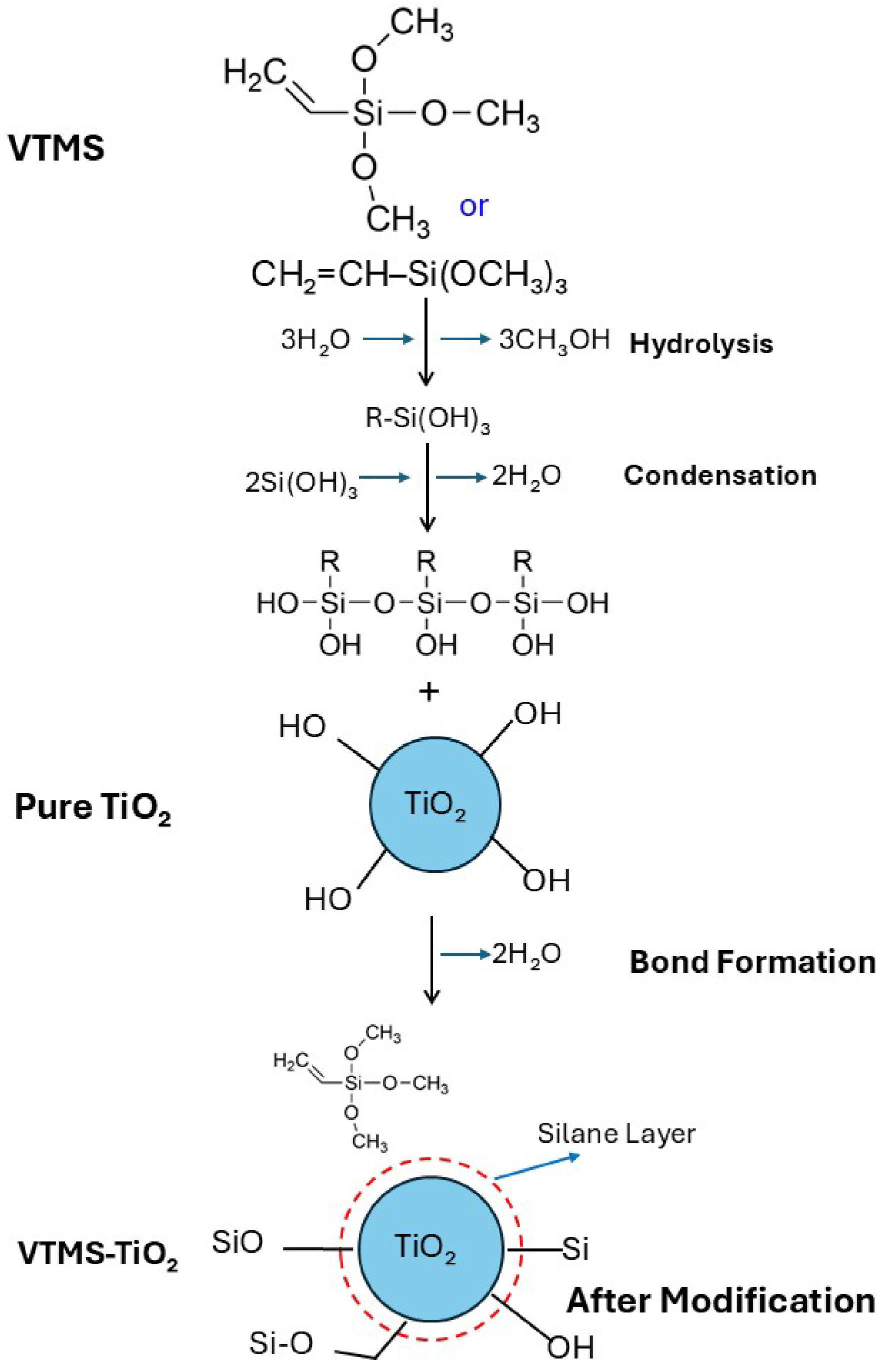

Figure S1: Schematic illustration of TiO_2 modification by VTMS

Table S3: Structures of chemicals used in this study

Sr. No.	Chemical	Structure
1	**Polysulfone (PSF)**	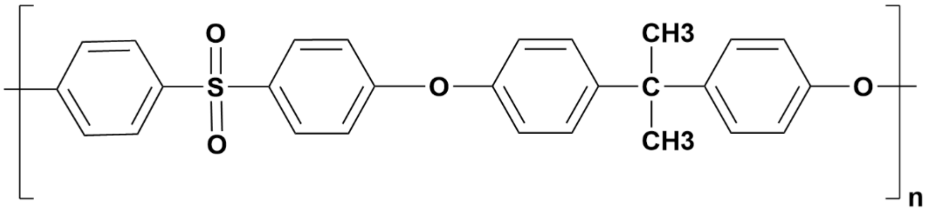
2	**N-methyl-2-pyrrolidone (NMP)**	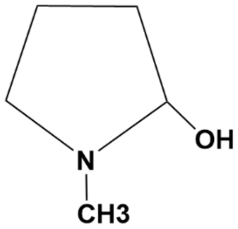
3	**Vinyl trimethoxy silane (VTMS)**	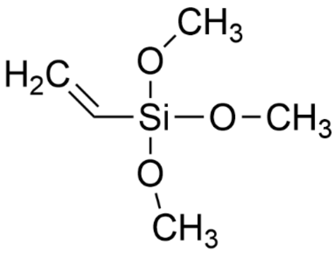

References

51. Maqsood, K.; Jamil, A.; Ahmed, A.; Sutisna, B.; Nunes, S.; Ulbricht, M. Effect of TiO2 on Thermal, Mechanical, and Gas Separation Performances of Polyetherimide–Polyvinyl Acetate Blend Membranes. Membranes 2023, 13, 734.

53. Zhu, H.; Yuan, J.; Zhao, J.; Liu, G.; Jin, W. Enhanced CO2/N2 separation performance by using dopamine/polyethyleneimine-grafted TiO2 nanoparticles filled PEBA mixed-matrix membranes. Sep. Purif. Technol. 2019, 214, 78-86. doi:https://doi.org/10.1016/j.seppur.2018.02.020.

18. Shamsabadi, A.A.; Seidi, F.; Salehi, E.; Nozari, M.; Rahimpour, A.; Soroush, M. Efficient CO2-removal using novel mixed-matrix membranes with modified TiO2 nanoparticles. J. Mater. Chem. A 2017, 5, 4011–4025.

50. Moghadam, F.; Omidkhah, M.R.; Vasheghani-Farahani, E.; Pedram, M.Z.; Dorosti, F. The effect of TiO2 nanoparticles on gas transport properties of Matrimid5218-based mixed matrix membranes. Sep. Purif. Technol. 2011, 77, 128–136. https://doi.org/10.1016/j.seppur.2010.11.032.

54. Sutrisna, P.D.; Savitri, E.; Gunawan, M.A.; Putri, I.H.F.; de Rozari, S.G.B. Synthesis, characterization, and gas separation performances of polysulfone and cellulose acetate-based mixed matrix membranes. Polym.-Plast. Technol. Mater. 2020, 59, 1300-1307. doi:10.1080/25740881.2020.1738471.
